# Repetitive Low-Level Blast Exposure via Akt/NF-κB Signaling Pathway Mediates the M1 Polarization of Mouse Alveolar Macrophage MH-S Cells

**DOI:** 10.3390/ijms241310596

**Published:** 2023-06-25

**Authors:** Chenhao Geng, Xinyue Wang, Jiale Chen, Na Sun, Yuru Wang, Zizheng Li, Lu Han, Shike Hou, Haojun Fan, Ning Li, Yanhua Gong

**Affiliations:** 1 Institute of Disaster and Emergency Medicine, Medical College, Tianjin University, Tianjin 300072, China; gengchenhao@tju.edu.cn (C.G.); wxy435014@tju.edu.cn (X.W.); 2019435009@tju.edu.cn (J.C.); snturbo@tju.edu.cn (N.S.); wyr@tju.edu.cn (Y.W.); lizz@tju.edu.cn (Z.L.); stu_hanlu@tju.edu.cn (L.H.); houshike@tju.edu.cn (S.H.); fanhj@tju.edu.cn (H.F.); 2 Tianjin Key Laboratory of Disaster Medicine Technology, Tianjin 300072, China

**Keywords:** repetitive low-level blast, Akt/NF-κB signaling pathway, macrophage polarization, early apoptosis, inhibitor, immune homeostasis

## Abstract

Repetitive low-level blast (rLLB) exposure is a potential risk factor for the health of soldiers or workers who are exposed to it as an occupational characteristic. Alveolar macrophages (AMs) are susceptible to external blast waves and produce pro-inflammatory or anti-inflammatory effects. However, the effect of rLLB exposure on AMs is still unclear. Here, we generated rLLB waves through a miniature manual Reddy-tube and explored their effects on MH-S cell morphology, phenotype transformation, oxidative stress status, and apoptosis by immunofluorescence, real-time quantitative PCR (qPCR), western blotting (WB) and flow cytometry. Ipatasertib (GDC-0068) or PDTC was used to verify the role of the Akt/NF-κB signaling pathway in these processes. Results showed that rLLB treatment could cause morphological irregularities and cytoskeletal disorders in MH-S cells and promote their polarization to the M1 phenotype by increasing iNOS, CD86 and IL-6 expression. The molecular mechanism is through the Akt/NF-κB signaling pathway. Moreover, we found reactive oxygen species (ROS) burst, Ca^2+^ accumulation, mitochondrial membrane potential reduction, and early apoptosis of MH-S cells. Taken together, our findings suggest rLLB exposure may cause M1 polarization and early apoptosis of AMs. Fortunately, it is blocked by specific inhibitors GDC-0068 or PDTC. This study provides a new treatment strategy for preventing and alleviating health damage in the occupational population caused by rLLB exposure.

## 1. Introduction

Explosions frequently occur in modern warfare, terrorist incidents, and chemical plants or gas leakage accidents, often resulting in a great number of casualties [1,2]. The explosive materials almost instantly transform from a solid or liquid state to a gas state after detonation and spread outward from the explosion point to produce a blast overpressure (BOP). Vast amounts of energy are transmitted to the surrounding medium, thus forming the blast wave [3,4,5]. People often pay more attention to organ injuries directly caused by high-level blast (HLB) waves (primary blast injury) or secondary injury. However, soldiers, rescuers, and other groups of people are often exposed to repetitive low-level blast (rLLB) waves for a long time [6]. It is worthy of in-depth investigation whether rLLB exposure has potential harm to the body. Current research on the harm of rLLB exposure is increasing, mainly focusing on its effects on the nervous system and tinnitus [7,8,9]. The blast waves can exert force on the air–tissue interfaces within the body, and the air-containing organs (such as the lung, auditory organs, and intestine) are vulnerable to the blast waves [3]. Among them, the lung, as the central place for gas exchange, may be particularly susceptible to blast waves. Different from HLB waves, rLLB waves may not cause direct damage to the alveolar tissues, but may lead to changes in pulmonary immune homeostasis [10,11].

Although at a steady state, alveolar epithelial cells occupy the majority of the lung surface. However, the immune network in the lung is highly diverse, including alveolar macrophages (AMs), parenchymal macrophages, lymphocytes, etc. At a steady state, the numbers of these immune cells are very small. However, under conditions of lung injury, allergy, pathogen exposure, etc., the number and diversity of cells can increase exponentially within hours to days [12]. AMs, as the main immune cells residing in the alveoli, play an important role in maintaining airway immune homeostasis [13,14]. However, studies on the effects of rLLB exposure on the phenotype transformation of AMs have not been reported.

Macrophages are very sensitive to external stimuli. Macrophages are characterized by high plasticity and can exert their role in modulating the immune response by rapidly changing their phenotype when they are exposed to different stimuli or microenvironments [15]. In 2000, Charlie Mills proposed a simple M1/2 macrophage phenotype paradigm of classically activated M1 phenotype (pro-inflammatory) and alternatively activated M2 phenotype (anti-inflammatory) to encapsulate the two contrasting functions played by macrophages in the regulation of inflammation [16,17]. M1 macrophages are largely involved in the inflammatory response and reactive oxygen species (ROS)-induced tissue injury and secrete a large amount of pro-inflammatory cytokines, such as interleukin (IL)-6, IL-1β and tumor necrosis factor (TNF)-α, while the M1 phenotype biomarkers inducible nitric oxide synthase (iNOS) and CD86 are highly expressed [18,19]. M2 macrophages have tissue repair and pro-vascular regenerative effects and mainly express anti-inflammatory factors such as IL-10 and transforming growth factor (TGF)-β, while the M2 phenotype biomarkers arginase 1 (Arg1) and CD206 are highly expressed [20]. This process of macrophage activation into M1/2 phenotype is defined as “macrophage polarization” [21]. The core function of macrophages is to sense the changes in the microenvironment and respond to the needs of organs through phenotypic transformation, to maintain homeostasis [22]. 

The widespread Akt pathway regulates proliferation, differentiation, and apoptosis by controlling numerous downstream target proteins [23,24]. NF-κB, a downstream target protein of Akt, is a signaling molecule essential for pulmonary inflammation [25]. It also plays a vital role in macrophage phenotype transformation [26,27,28,29]. However, it is unclear whether the Akt/NF-κB signaling pathway is activated in AMs after rLLB exposure.

Therefore, this study aimed to investigate the effects of rLLB exposure on the phenotype of AMs and the role of the Akt/NF-κB signaling pathway in them. We first generated the rLLB waves through a miniature manual Reddy-tube, and then explored their effects on the morphology, phenotype transformation, oxidative stress status, and apoptosis of MH-S cells. Finally, the role of the Akt/NF-κB signaling pathway was confirmed by inhibitors of Akt and NF-κB, respectively. This study is expected to provide potential therapeutic strategies for preventing and mitigating the hazards of rLLB exposure for occupational populations.

## 2. Results

### 2.1. rLLB Exposure Caused Morphological Changes and Polarization toward M1 Phenotype of MH-S Cells

**Figure 1:**
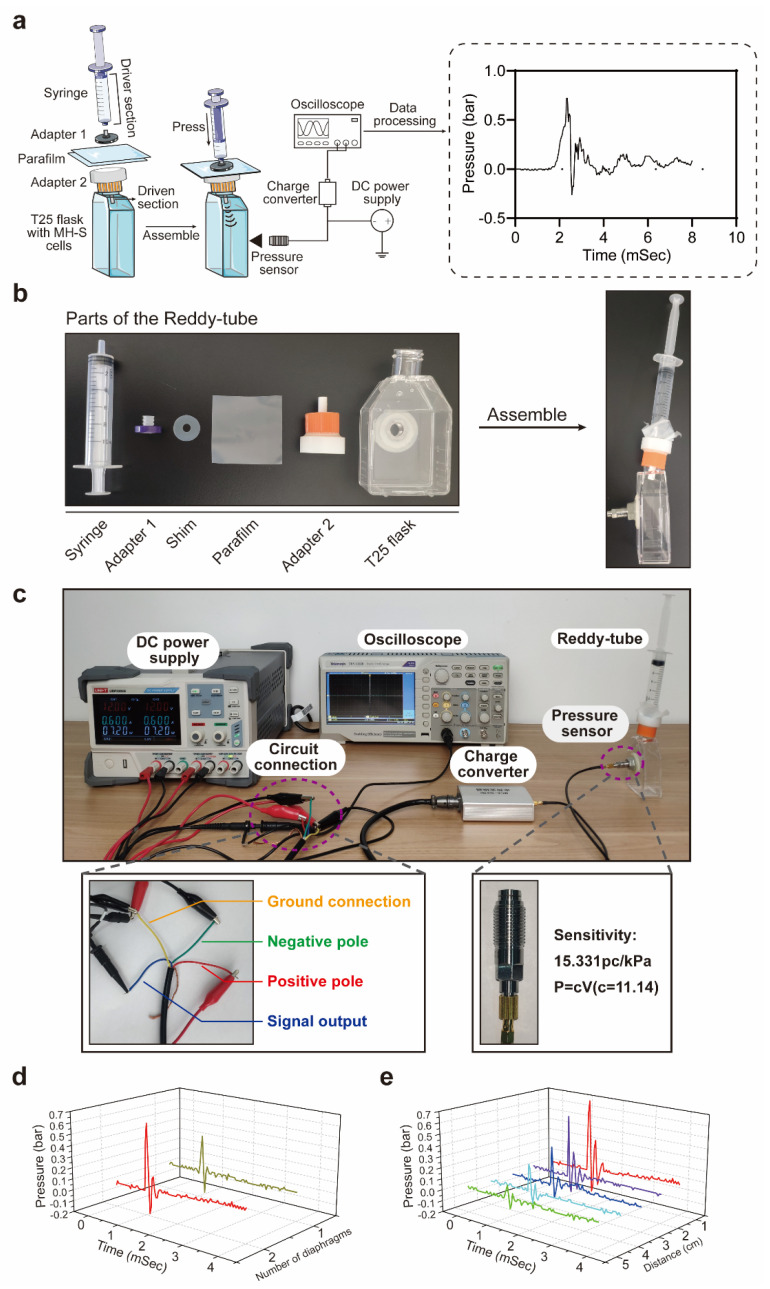
Miniature manual Reddy-tube device. (**a**) Schematic diagram of the miniature manual Reddy-tube device and the real waveform of two Parafilm M membranes. (**b**) Physical assembly diagram of the miniature manual Reddy-tube device. (**c**) Scene diagram of shock waveform detection. (**d**) Relationship between the diaphragm number and the pressure curves. Red and green lines represent the double and single diaphragms, separately. (**e**) Relationship between the distance and the peak overpressure in the double diaphragm. The horizontal axis 0 points of (**d**,**e**) are normalized to the first 1 ms of the overpressure signal generation.

In order to investigate the effects of rLLB exposure on AMs, we assembled a miniature manual Reddy-tube device as shown in Figure 1a–c. We detected the relationship between the diaphragm number and the pressure curves. The results showed that the peak overpressure of single and double diaphragms was 0.38 bar and 0.62 bar, separately (Figure 1d). To generate higher pressures, we chose double diaphragms for subsequent experiments. Meanwhile, within the length of the T25 flask, we also obtained the relationship between peak overpressure and distance (Figure 1e). The results indicated that the peak overpressure gradually decreased with increasing distance. However, the peak overpressure was still present at the farthest position of the T25 flask.

**Figure 2:**
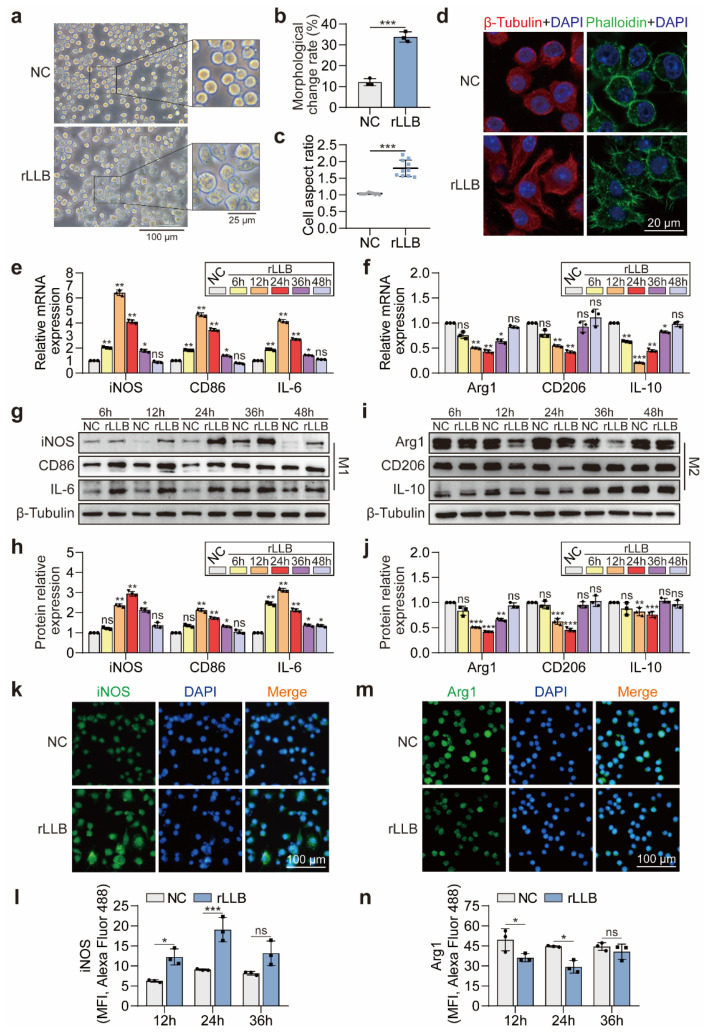
Effect of rLLB exposure on morphological and phenotype transformation of MH-S cells. (**a**) Optical microscope images of MH-S cells in NC and rLLB group at 6h. The **right** panel (scale bar: 25 μm) is a local magnification of the **left** panel (scale bar: 100 μm). (**b**,**c**) The statistics of the cell morphology change rate and cell aspect ratio in the graphs of (**a**) (*n* = 10). (**d**) Confocal microscopy images of cells in NC and rLLB groups stained for nuclei (DAPI, blue), β-Tubulin (Red), and Phalloidin (green) to detect cytoskeletal changes (scale bars: 20 μm). (**e**–**g**,**i**) qPCR and WB analysis of the expression of M1 biomarkers iNOS, CD86, IL-6 and M2 biomarkers Arg1, CD206, IL-10 at 6 h, 12 h, 24 h, 36 h, 48 h in NC and rLLB groups. (**h**,**j**) Quantification of the grayscale values of (**g**,**i**), respectively (*n* = 3). (**k**,**m**) Immunofluorescence analysis of the iNOS and Arg1 expression in MH-S cells at 24 h (scale bar: 100 μm). (**l**,**n**) Quantification of the mean fluorescence intensity (MFI) of iNOS and Arg1 expression at 12 h, 24 h, and 36 h, respectively (*n* = 3). Data are presented as the means ± SD. The NC groups at different time points in (**e**,**f**,**h**,**j**) are normalized to 1, respectively. (**b**,**c**) Using unpaired *t* test. (**e**,**f**,**h**,**j**,**l**,**n**) Using two-way ANOVA followed by Sidak’s multiple comparisons test. * *p* < 0.05, ** *p* < 0.01, *** *p* < 0.001, ns: no significance.

Then, we applied rLLB waves to MH-S cells via a miniature manual Reddy-tube device. In terms of morphology, we observed under the optical microscope that the cells in the NC group had regular morphology and showed shiny round shapes. However, the cells in the rLLB group had irregular morphology and extended pseudopods (Figure 2a). Compared with the NC group, the proportion of morphologically irregular cells was significantly upregulated in the rLLB group (Figure 2b, 12.07 ± 1.75% vs. 33.8 ± 2.42%, *p* = 0.0004), and the aspect ratio was significantly increased (Figure 2c, 1.04 ± 0.02 vs. 1.80 ± 0.25, *p* < 0.0001). As important components of the cytoskeleton, microfilaments and microtubules play a crucial role in maintaining cell morphology. Therefore, we assessed the effect of rLLB exposure on the cytoskeleton by fluorescently labeling microfilaments and microtubules. Microtubules are composed of α- and β-tubulin heterodimers, in which β-tubulin was labeled in red (β-Tubulin-Alexa Fluor 488). Microfilaments are composed of actin polymers that were labeled in green (Phalloidin-FITC) (Figure 2d). Confocal microscopy observed that microtubules in the NC group were uniformly distributed in the cytoplasm in the form of curved thin filaments, and the microfilaments were regularly distributed around the cell membrane (Figure 2d, up), while in the rLLB group, microtubules were reorganized to form longer filaments extending outward, and microfilaments spread out from around the cell membrane to form burrs, giving the cell an irregular shape (Figure 2d, down). This showed that the rLLB exposure caused morphological changes and cytoskeletal disorders in MH-S cells.

The morphological changes in macrophages are closely related to their functional performance [30,31], so does rLLB exposure cause MH-S macrophage polarization? qPCR results showed that the expression levels of M1 macrophage-related molecules *iNOS*, *CD86*, and *IL-6* in the rLLB group were significantly upregulated at 6 h, 12 h, 24 h, and 36 h compared with the NC group, and all reached the maximum at 12 h. The maximum values of *iNOS*, *CD86*, and *IL-6* in the rLLB group were 6.43 ± 0.22-fold (*p* = 0.0028), 4.67 ± 0.15-fold (*p* = 0.0028) and 4.16 ± 0.12-fold (*p* = 0.0024) higher than those in the NC group, respectively. However, at 48 h, there was no significant difference in the expression of these three molecules compared to the NC group (Figure 2e). The expression of M2 macrophage-related molecule *Arg1* was significantly downregulated in the rLLB group at 12 h, 24 h, and 36 h and reached a minimum value at 24 h. The minimum value was 0.43 ± 0.03-fold lower than that in the NC group (*p* = 0.0043). The expression of *CD206* was significantly downregulated at 12 h, 24 h, and reached a minimum value at 24 h. The minimum value was 0.42 ± 0.02-fold lower than that in the NC group (*p* = 0.0021). *IL-10* expression was significantly downregulated at 6 h, 12 h, 24 h, 36 h and reached a minimum value at 12 h. The minimum value was 0.21 ± 0.01-fold lower than that in the NC group (*p* = 0.0002) (Figure 2f). WB assays obtained similar results with qPCR (Figure 2g–i). Grayscale analysis of WB bands showed that the expression of iNOS, CD86, and IL-6 increased significantly at 6 h, 12 h, 24 h, and 36 h in the rLLB group compared with the NC group. iNOS expression reached its maximum value at 24 h, 2.94 ± 0.12-fold higher than that in the NC group (*p* = 0.0007). CD86 expression reached a maximum value at 12 h, 2.12 ± 0.09-fold higher than that in the NC group (*p* = 0.0099). IL-6 expression reached a maximum value at 24 h, 2.10 ± 0.07-fold higher than that in the NC group (*p* = 0.0027) (Figure 2h). There was a significant downregulation of Arg1, CD206, and IL-10 expression at 12 h and 24 h, and all reached a minimum value at 24 h. Arg1, CD206, and IL-10 expression levels were 0.42 ± 0.01-fold (*p* = 0.0001), 0.46 ± 0.04-fold (*p* < 0.0001) and 0.76 ± 0.06-fold (*p* < 0.0002) lower than those in the NC group, respectively (Figure 2j). Immunofluorescence staining of iNOS and Arg1 also obtained similar results with WB (Figure 2k,m). There was an increasing trend in iNOS expression at 12 h and 24 h, reaching a maximum value at 24 h (Figure 2l, 9.04 ± 0.19 vs. 19.05 ± 3.02, *p* = 0.0005). Moreover, a decreasing trend in Arg1 expression at 12 h and 24 h, reaching a minimum value at 24 h (Figure 2n, 44.64 ± 0.52 vs. 29.34 ± 4.68, *p* = 0.0209).

In summary, the rLLB treatment increased the expression of M1 macrophage molecular markers and decreased the expression of M2 macrophage molecular markers in MH-S cells, indicating that rLLB exposure could convert AMs to the M1 phenotype.

### 2.2. rLLB Exposure Induced Oxidative Stress State Changes and Early Apoptosis of MH-S Cells

**Figure 3:**
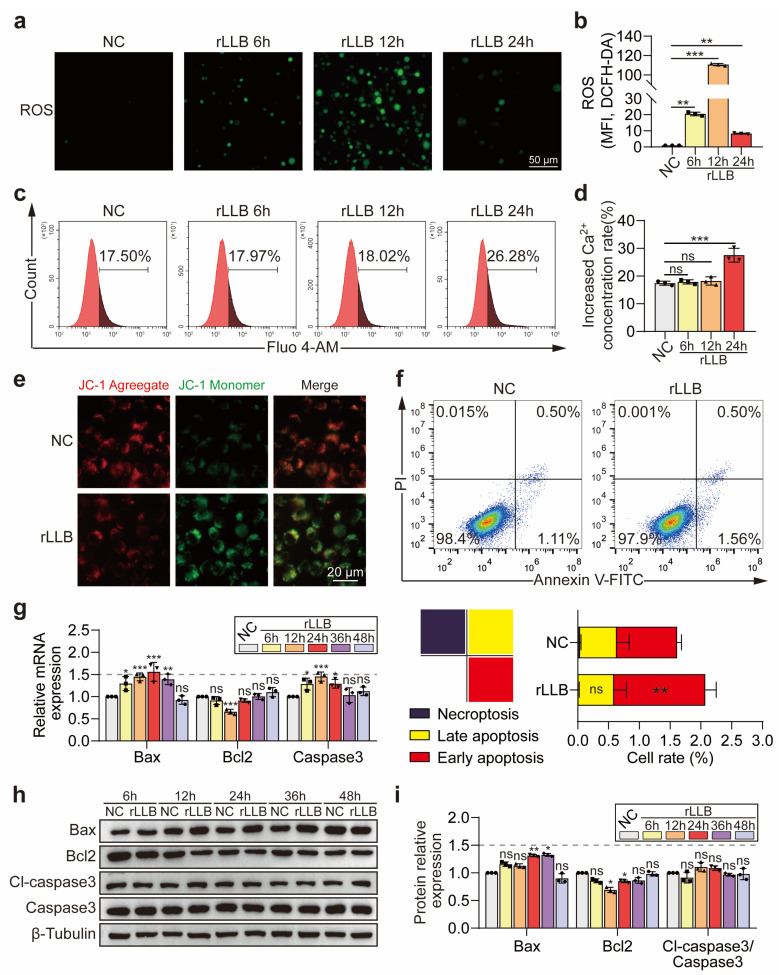
Effects of rLLB exposure on oxidative stress state and early apoptosis in MH-S cells. (**a**) Immunofluorescence analysis of ROS (DCFH-DA fluorescent labeling) expression in the NC and rLLB groups at 6h, 12h, and 24h (scale bar: 50 μm). (**b**) Quantification of the mean fluorescence intensity (MFI) of the image in (**a**). (**c**) Flow cytometry analysis of the Ca^2+^ accumulation by Fluo-4 AM in NC and rLLB groups at 6 h, 12 h, and 24 h. (**d**) Quantification of the Ca^2+^ ratio in (**c**). (**e**) Immunofluorescence analysis of the mitochondrial membrane potential changes by JC-1 staining in the NC and rLLB groups at 24 h. JC-1 aggregates (red) represent high membrane potential. JC-1 monomers (green) represent low membrane potential (scale bar: 20 μm). (**f**) Flow cytometry analysis of MH-S cell apoptosis in the NC and rLLB groups at 24 h by Annexin V/PI double staining. (**g**,**h**) qPCR and WB analysis of the expression of apoptosis-related molecules Bax, Bcl2, Caspase3, and Cleaved-caspase3 at 6 h, 12 h, 24 h, 36 h, and 48 h. (**i**) Quantification of the grayscale values of (**h**). Data are presented as the means ± SD (*n* = 3). The NC groups at different time points in (**g**,**i**) are normalized to 1, respectively. (**b**,**d**,**g**,**i**) Using two-way ANOVA followed by Sidak’s multiple comparisons tests. (**f**) Using unpaired *t*-test. * *p* < 0.05, ** *p* < 0.01, *** *p* < 0.001, ns: no significance.

The M1 pro-inflammatory phenotype of macrophages is often accompanied by a significant increase in ROS level [32,33]. To investigate the effect of rLLB exposure on the oxidative stress status of MH-S cells, we detected the changes in intracellular reactive oxygen levels by ROS probe (DCFH-DA) at 6 h, 12 h, and 24 h. The results indicated that there was a significant burst of ROS in the cells of the rLLB group at 6 h, 12 h, and 24 h compared with the NC group (Figure 3a). The maximum value occurred at 12 h, which was 110.65 ± 1.15-fold higher than that in the NC group (Figure 3b, *p* = 0.0001). Cellular oxidative stress status is often associated with endoplasmic reticulum stress and mitochondrial dysfunction-mediated apoptosis [34,35], so we used the Ca^2+^ probe Fluo-4 AM to detect the intracellular Ca^2+^ concentration by flow cytometry (Figure 3c,d). The results indicated that there was no significant difference in intracellular Ca^2+^ concentration at 6 h and 12 h in the rLLB group compared with the NC group, while there was a significant upregulation at 24 h (Figure 3c,d, *p* = 0.0001), suggesting that Ca^2+^ release due to endoplasmic reticulum stress may have occurred after the ROS burst. We then examined the mitochondrial membrane potential (ΔΨm) of MH-S cells by JC-1 at 24 h. The decrease in ΔΨm prevented the accumulation of JC-1 in mitochondria and promoted its dispersion throughout the cell, leading to the transfer of JC-1 aggregates (red fluorescence) to JC-1 monomers (green fluorescence). The results showed that the green fluorescence was significantly increased in the rLLB group compared with that in the NC group (Figure 3e), indicating that the rLLB treatment led to a decrease in mitochondrial membrane potential in MH-S cells, resulting in mitochondrial dysfunction. The decrease in mitochondrial membrane potential is a hallmark event in the early stage of apoptosis. Therefore, we applied flow cytometry by Annexin V/PI double staining and found that early apoptosis of the cells at 24 h in the rLLB group was significantly different compared with that in the NC group, while there was no significant difference in late apoptosis and necrotic apoptosis (Figure 3f). Subsequently, the changes in apoptosis-related molecules Bax, Bcl2, and Caspase3 were detected at the mRNA and protein levels. qPCR results suggested that the pro-apoptotic gene *Bax* expression was slightly upregulated in the rLLB group compared with the NC group at 24 h, which was 1.56 ± 0.21-fold higher than that in the NC group (*p* < 0.0001). The expression of apoptosis suppressor gene *Bcl2* was downregulated and reached the minimum value at 12 h, which was 0.66 ± 0.06-fold lower than that in the NC group (*p* < 0.0001) and was not significantly different between the NC and rLLB groups at 6 h, 24 h, 36 h, and 48 h. *Caspase3* expression was slightly upregulated at 6 h, 12 h, and 24 h, reaching a maximum value at 12 h, but the maximum value was 1.45 ± 0.10-fold higher than that in the NC group (*p* = 0.0001), not reaching the 1.5-fold that is usually considered a biologically significant difference, and not significantly different at 36 h and 48 h (Figure 3g). WB results showed that Bax was slightly upregulated in the rLLB group compared with the NC group at 24 h and 36 h. The maximum value appeared at 36 h, which was 1.33 ± 0.03-fold higher than that in the NC group (*p* = 0.0109), but did not reach a 1.5-fold biologically significant difference, and no significant difference was observed at 6 h, 12 h, 48 h (Figure 3h,i). Bcl2 expression was downregulated at 12 h and 24 h compared with the NC group. It reached a minimum value at 12 h, which was 0.69 ± 0.05-fold that of of NC group (*p* = 0.0380), and Bcl2 expression was not significantly different from that in the NC group at 6 h, 36 h, and 48 h. The ratio of functional Cleaved-caspase3 to precursor Caspase3 was not significantly different between the NC and rLLB groups (Figure 3h,i). In other words, there were few changes in late apoptosis-related molecules.

The above results demonstrated that rLLB exposure caused changes in oxidative stress status and mild early apoptosis in AMs. This also shows to a certain extent that rLLB is more likely to change immune homeostasis than to cause a large number of cell deaths.

### 2.3. rLLB Exposure Activated the Akt/NF-κB Signaling Pathway in MH-S Cells

**Figure 4:**
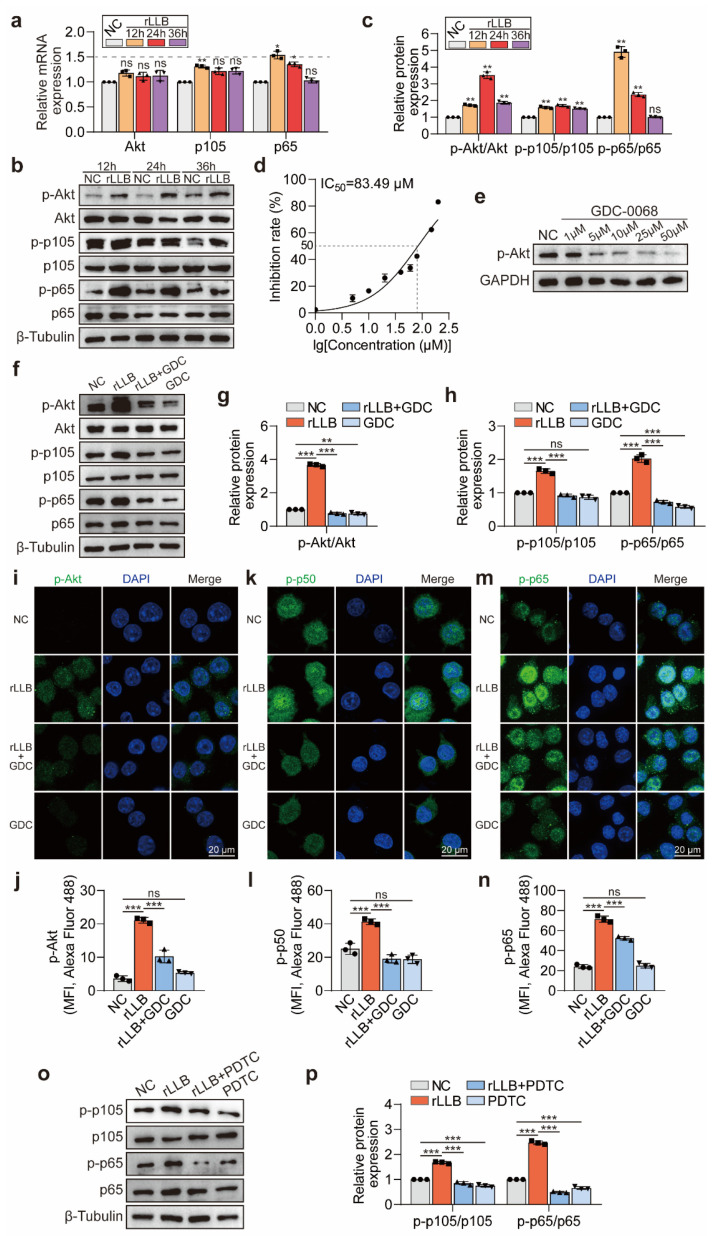
Activation of Akt/NF-κB signaling pathway in MH-S cells induced by rLLB exposure. (**a**) qPCR analysis of Akt/NF-κB signaling pathway-related molecules *Akt*, *p105*, and *p65* expression at 12 h, 24 h, and 36 h in the NC and rLLB groups. (**b**) WB analysis of p-Akt, Akt, p-p105, p105, p-p65, and p65 expression at 12 h, 24 h, and 36 h in the NC and rLLB groups at the protein level. (**c**) Quantification of the grayscale value of (**b**). (**d**) Inhibition rate of MH-S cell growth induced by treatment with 1 μM, 5 μM, 10 μM, 20 μM, 40 μM, 60 μM, 80 μM, 150 μM, and 200 μM GDC-0068 for 12 h by CCK-8 assay. (**e**) WB analysis of p-Akt expression at different concentrations of GDC-0068 (1 μM, 5 μM, 10 μM, 25 μM, and 50 μM) for 12 h. (**f**) WB analysis of the effect of adding 10 μM GDC-0068 on p-Akt, Akt, p-p105, p105, p-p65, and p65 expression in MH-S cells at 24 h. (**g**,**h**) Quantification of the grayscale values of (**f**). (**i**,**k**,**m**) Immunofluorescence analysis of the expression of p-Akt, p-p50, and p-p65 in four groups at 24 h. (**j**,**l**,**n**) Quantification of MFI in (**i**,**k**,**m**), respectively. (**o**) WB analysis of the effect of adding 20 μM PDTC on the expression of p-p105, p105, p-p65, and p65 at 24 h. (**p**) Quantification of the grayscale values of (**o**). Data are presented as the means ± SD (*n* = 3). The NC groups at different time points in (**a**,**c**) are normalized to 1, respectively. (**a**,**c**) Using two-way ANOVA followed by Sidak’s multiple comparisons tests. (**g**,**h**,**j**,**l**,**n**,**p**) Using one-way ANOVA followed by Tukey’s multiple comparisons tests. * *p* < 0.05, ** *p* < 0.01, *** *p* < 0.001, ns: no significance.

Previous research indicated that the Akt/NF-κB signaling pathway played a vital role in macrophage polarization [25]. To determine whether the Akt/NF-κB signaling pathway was activated in MH-S cells treated with rLLB exposure, we first detected the expression of Akt/NF-κB signaling pathway-associated molecules, including Akt, p105, and p65. qPCR results showed that the *Akt* and *p105* expression in the rLLB group was not biologically significantly different at 12 h, 24 h, and 36 h compared with the NC group (Figure 4a). The *p65* expression was different from that in the NC group at 12 h and 24 h, reaching a maximum value at 12 h, which was 1.54 ± 0.08-fold that in the NC group (*p* = 0.0196), and not significantly different at 36 h (Figure 4a). Since the activation of the Akt/NF-κB signaling pathway was mainly reflected by the increase in Akt, p105, and p65 protein phosphorylation levels, we detected the changes in p-Akt, Akt, p-p105, p105, p-p65, and p65 protein levels by WB (Figure 4b). The grayscale analysis showed that the ratios of p-pAkt/Akt, p-105/p105, and p-p65/p65 were significantly upregulated in the rLLB group compared with the NC group. The ratios of p-pAkt/Akt and p-105/p105 all reached maximum values at 24 h, which were 3.52 ± 0.20-fold (*p* = 0.0065) and 1.70 ± 0.07-fold (*p* = 0.0096) higher than those in the NC group, respectively. P-p65/p65 reached a maximum value at 12 h, which was 4.92 ± 0.33-fold higher than that in the NC group (*p* = 0.0068) (Figure 4c). To further verify the role of the Akt/NF-κB signaling pathway in the polarization of MH-S cells induced by rLLB exposure, we applied molecular inhibitors to block this signaling pathway and explored its effects on the downstream signaling pathway. GDC-0068 is a highly selective pan-Akt inhibitor that specifically inhibits Akt. The IC_50_ value of GDC-0068 for MH-S cells obtained by CCK8 assay was 83.49 μM (Figure 4d). Using a concentration gradient of GDC-0068 below the IC_50_ value (1 μM, 5 μM, 10 μM, 25 μM, 50 μM) to treat MH-S cells, WB results showed that p-Akt expression could be significantly inhibited by 5 μM, 10 μM, 25 μM, and 50 μM GDC-0068 (Figure 4e). Meanwhile, a 10 μM concentration had already achieved an inhibitory effect comparable to that of 25 μM and 50 μM. Considering the IC_50_ value of GDC-0068 for MH-S cells, 10 μM was selected as the working concentration, which was relatively safe for cell growth.

We subsequently added 10 μM GDC-0068 to MH-S cells after treatment with rLLB. WB results indicated that the p-Akt expression in the rLLB group was 3.66 ± 0.08-fold that in the NC group, while in the rLLB + GDC group, the expression was downregulated to 0.77 ± 0.06-fold that in the NC group (Figure 4f,g, *p* < 0.0001). The expression of p-p105 was downregulated from 1.65 ± 0.07-fold in the rLLB group to 0.92 ± 0.05-fold in the rLLB + GDC group, and the expression of p-p65 was downregulated from 2.03 ± 0.12-fold in the rLLB group to 0.73 ± 0.05-fold in the rLLB + GDC group (Figure 4f,h, *p* < 0.0001). This indicated that Akt inhibitor GDC-0068 could significantly inhibit the upregulation of p-Akt, p-p105, and p-p65 expression induced by rLLB exposure. Similarly to the WB results, immunofluorescence of p-Akt, p-p50, and p-p65 showed the same trend, and p-p50 and p-p65 translocated to the nucleus in the rLLB group (Figure 4i–n). We also applied the NF-κB inhibitor PDTC, and WB results also demonstrated that PDTC inhibited the upregulation of p-p105 and p-p65 expression induced by rLLB exposure (Figure 4o,p).

The above results suggested that rLLB exposure could induce the activation of the Akt/NF-κB signaling pathway in AMs.

### 2.4. Inhibition of the Akt/NF-κB Signaling Pathway Downregulated MH-S Cell Phenotypic Changes Induced by rLLB Exposure

**Figure 5:**
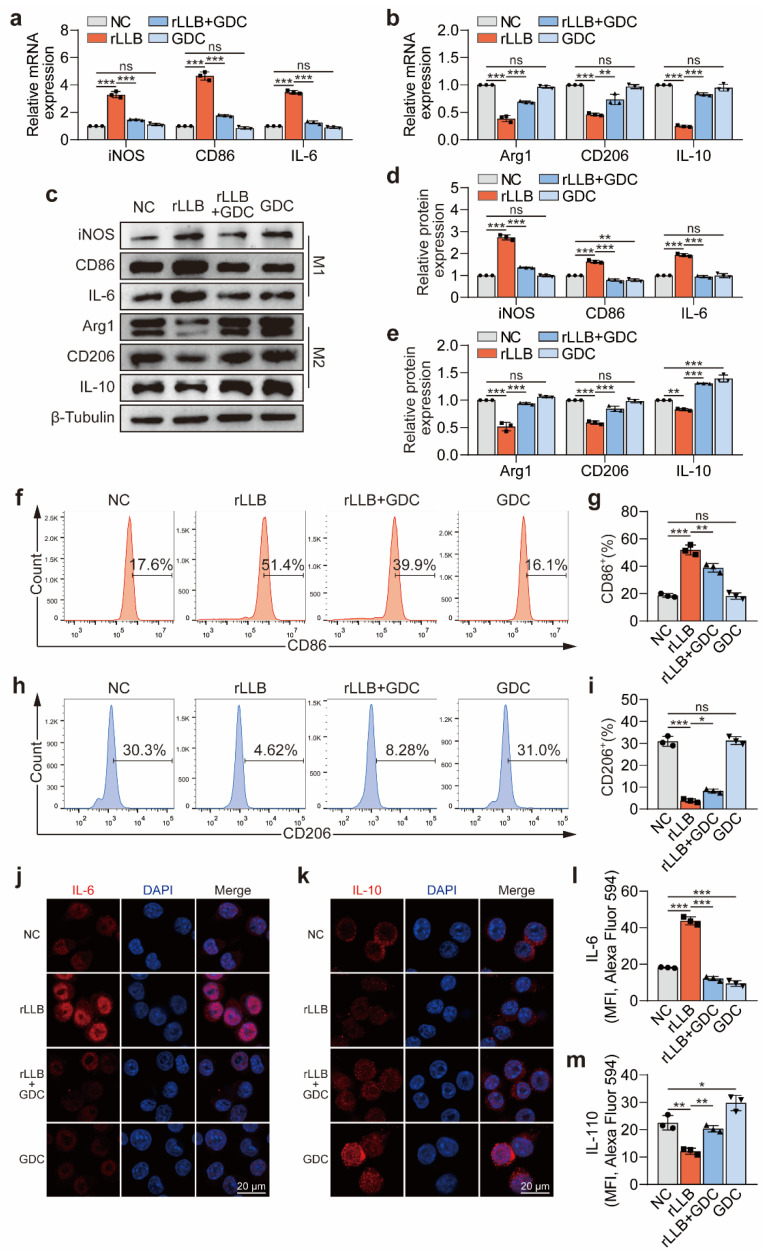
Effect of GDC-0068 on the phenotype transformation of MH-S cells induced by rLLB exposure. (**a**–**c**) qPCR and WB analysis of the effect of adding GDC-0068 (10 μM) on iNOS, CD86, IL-6, Arg1, CD206, and IL-10 expression in MH-S cells at 24 h. (**d**,**e**) Quantification of the grayscale values of (**c**). (**f**,**h**) Flow cytometry analysis of the effect of adding GDC-0068 (10 μM) on the ratio of M1/M2 macrophages at 24 h by CD86 and CD206 labeling. (**g**,**i**) Quantification of the CD86 or CD206 positive cells proportion of (**f**,**h**), respectively. (**j**,**k**) Immunofluorescence analysis of the effect of adding GDC-0068 (10 μM) on the expression of IL-6 and IL-10 in MH-S cells at 24 h. (**l**,**m**) Quantification of MFI in (**j**,**k**), respectively. Data are presented as the means ± SD (*n* = 3). All statistics in this figure use one-way ANOVA followed by Tukey’s multiple comparisons tests. * *p* < 0.05, ** *p* < 0.01, *** *p* < 0.001, ns: no significance.

To investigate the role of the Akt/NF-κB signaling pathway in the phenotypic changes in MH-S cells induced by rLLB waves, especially the effect of M1 polarization, we applied GDC-0068 to block the Akt/NF-κB signaling pathway. Compared with the NC group, qPCR results showed that *iNOS* expression was downregulated from 3.29 ± 0.23-fold in the rLLB group to 1.47 ± 0.04-fold in the rLLB + GDC group, the expression of *CD86* was downregulated from 4.66 ± 0.30-fold in the rLLB group to 1.77 ± 0.06-fold in the rLLB + GDC group, and the expression of *IL-6* was downregulated from 3.47 ± 0.13-fold in the rLLB group to 1.26 ± 0.11-fold in the rLLB + GDC group (Figure 5a, *p* < 0.0001). Meanwhile, compared with the NC group, *Arg1* expression was upregulated from 0.39 ± 0.06-fold in the rLLB group to 0.69 ± 0.02-fold in the rLLB + GDC group, *CD206* expression was upregulated from 0.46 ± 0.02-fold in the rLLB group to 0.73 ± 0.10-fold in the rLLB + GDC group, and *IL-10* expression was upregulated from 0.25 ± 0.02-fold in the rLLB group to 0.83 ± 0.03-fold in the rLLB + GDC group (Figure 5b, *p* < 0.0001). WB results revealed the same trend (Figure 5c–e). It indicated that GDC-0068 could significantly inhibit the upregulation of M1 macrophage-related molecules iNOS, CD86, and IL-6 expression and the downregulation of M2 macrophage-related molecules Arg1, CD206, and IL-10 expression induced by rLLB waves. That is, inhibition of the Akt/NF-κB signaling pathway could downregulate MH-S cell polarization toward the M1 phenotype.

**Figure 6:**
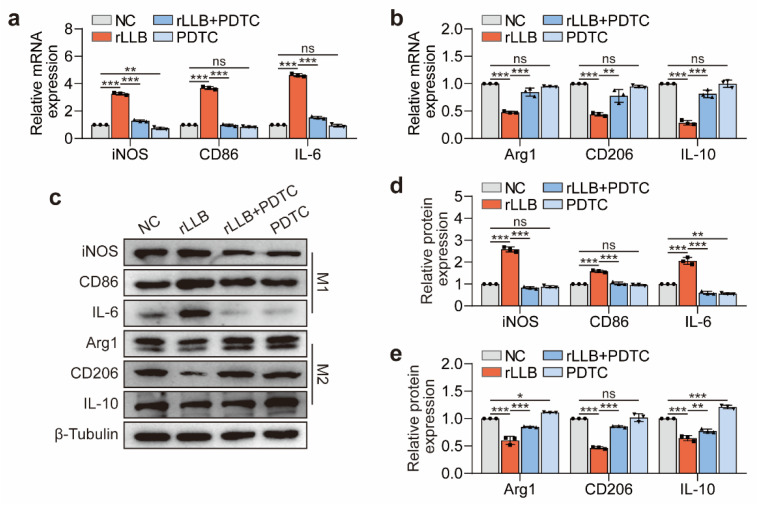
Effect of PDTC on the phenotype transformation of MH-S cells induced by rLLB exposure. (**a**–**c**) qPCR and WB analysis of the effect of adding PDTC (20 μM) on iNOS, CD86, IL-6, Arg1, CD206, and IL-10 expression in MH-S cells at 24 h. (**d**,**e**) Quantification of the grayscale values of (**c**). Data are presented as the means ± SD (*n* = 3). All statistics in this figure use one-way ANOVA followed by Tukey’s multiple comparisons tests. * *p* < 0.05, ** *p* < 0.01, *** *p* < 0.001, ns: no significance.

Subsequently, we used CD86 and CD206 to label M1 and M2 macrophages, respectively, and analyzed the effect of applying GDC-0068 to block the Akt/NF-κB signaling pathway on the M1/M2 macrophage ratio by flow cytometry. The results indicated that GDC-0068 could inhibit the upregulation of the M1 macrophage ratio (Figure 5f,g, *p* = 0.0017) and the downregulation of the M2 macrophage ratio induced by rLLB waves (Figure 5h,i, *p* = 0.0319). It was consistent with the qPCR and WB results. Immunofluorescence staining for the inflammatory cytokines IL-6 and IL-10 revealed that GDC-0068 significantly inhibited the upregulation of IL-6 expression and the downregulation of IL-10 expression induced by rLLB waves (Figure 5j–m, *p* < 0.0001). Meanwhile, we applied NF-κB inhibitor PDTC, which obtained similar results at mRNA and protein levels (Figure 6a–e).

The above results suggested that the inhibition of the Akt/NF-κB signaling pathway could reduce the phenotype transformation of AMs induced by rLLB exposure, especially toward the M1 phenotype.

### 2.5. Inhibition of the Akt/NF-κB Signaling Pathway Had No Significant Effect on Early Apoptosis of MH-S Cells Induced by rLLB Exposure

**Figure 7:**
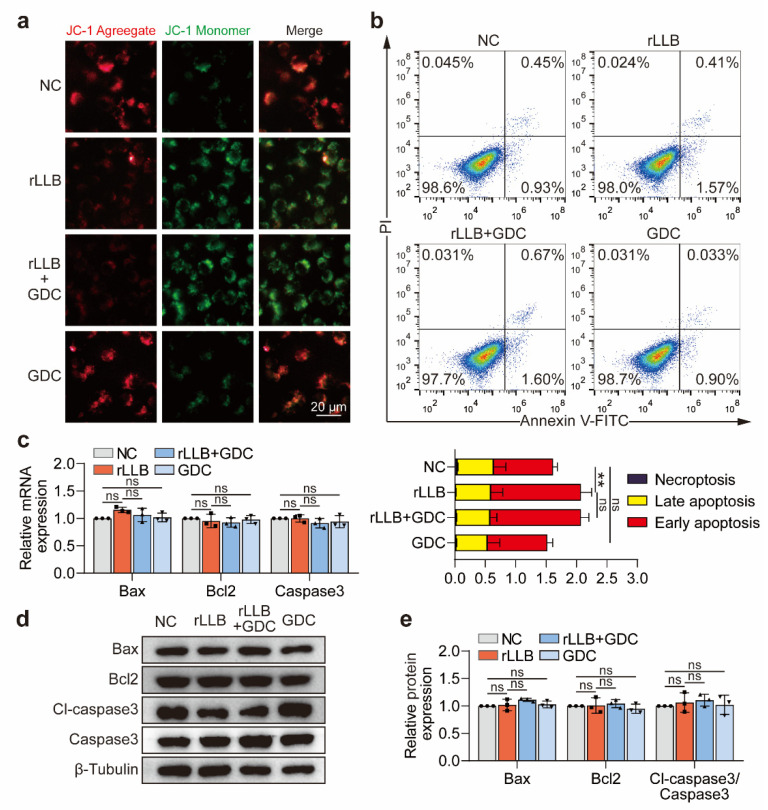
Effect of GDC-0068 on the early apoptosis of MH-S cells induced by rLLB exposure. (**a**) Immunofluorescence analysis of the mitochondrial membrane potential changes by JC-1 staining at 24 h (scale bar: 20 μm). (**b**) Flow cytometry analysis of MH-S cell apoptosis at 24 h by Annexin V/PI double staining. (**c**,**d**) qPCR and WB analysis of the expression of apoptosis-related molecules Bax, Bcl2, Caspase3, and Cleaved-caspase3. (**e**) Quantification of the grayscale values of (**d**). Data are presented as the means ± SD (*n* = 3). All statistics in this figure use one-way ANOVA followed by Tukey’s multiple comparisons tests. ** *p* < 0.01, ns: no significance.

We applied GDC-0068 to block the Akt/NF-κB signaling pathway to verify its effect on early apoptosis of MH-S cells induced by rLLB waves. However, the results showed that GDC-0068 was unable to upregulate the decreased mitochondrial membrane potential detected by JC-1 (Figure 7a). Meanwhile, GDC-0068 was unable to decrease the MH-S cells number of early apoptosis due to the rLLB exposure (Figure 7b). Similarly, GDC-0068 had no effect on the expression of apoptosis-related molecules (Figure 7c–e), probably because the early apoptosis caused by rLLB was relatively weak and had no significant effect on the late apoptosis.

The above results suggested that inhibition of the Akt/NF-κB signaling pathway had no significant effect on the early apoptosis of AMs induced by rLLB exposure.

## 3. Discussion

**Figure 8:**
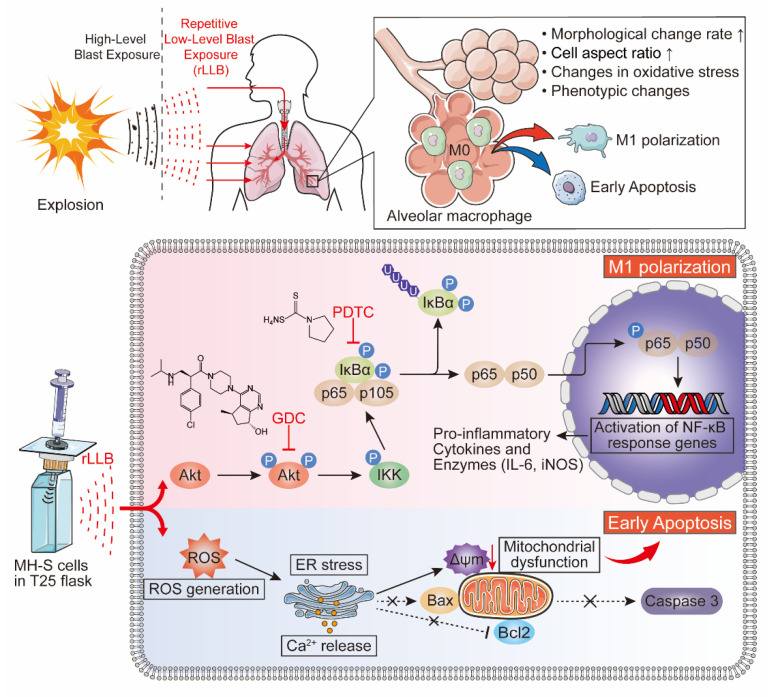
Schematic diagram of the mechanism of AM polarization toward M1 phenotype and early apoptosis induced by rLLB exposure. The blast waves can reach the lungs through the thorax or directly through the open airway, producing stimulation of AMs and causing M1 polarization and early apoptosis. The rLLB exposure generated by the Reddy-tube acted on MH-S cells, causing macrophage polarization to M1 phenotype through activation of the Akt/NF-κB signaling pathway. The rLLB exposure can also lead to ROS burst, Ca^2+^ accumulation, mitochondrial membrane potential abnormalities and early apoptosis. Highly selective pan-Akt inhibitor (GDC-0068) and NF-κB inhibitor (PDTC) could inhibit the Akt/NF-κB signaling pathway and decrease macrophage polarization to the M1 phenotype.

The results of this study demonstrated that rLLB exposure could cause AMs to polarize toward the M1 phenotype, and the polarization process was regulated by the Akt/NF-κB signaling pathway. Additionally, rLLB exposure could cause ROS burst, Ca^2+^ accumulation, mitochondrial membrane potential reduction, and early apoptosis in AMs (Figure 8).

The blast waves can reach the lungs through the thorax or directly through the open airway, dissipating the kinetic energy carried in the lung tissue [36] (Figure 8 upper left). As the central place where the body completes gas exchange, the alveoli have a large number of air–tissue interfaces, and given the tissue density gradient, the blast waves will cause alveolar traction [37]. Due to the low intensity of rLLB waves, exposure will not lead to the rupture of alveoli due to excessive traction. However, the pulmonary immune system is sensitive to external stimulation. Previous studies have provided evidence that the immune system can respond to mechanical stimulation [10,11], so the alveolar traction caused by rLLB waves is likely to lead to changes in pulmonary immune homeostasis. Among them, AMs, as the main resident immune cells in the alveoli, are likely to expand exponentially for hours to days after being stimulated by external forces. Therefore, our study first assessed the effect of rLLB exposure on the morphology of MH-S cells. The results demonstrated that there was an increase in the macrophage aspect ratio. Cells became more extended, and the cytoskeleton was disturbed. This phenomenon may be related to the external pulling effect or the polarization of macrophages [38]. McWhorter et al. showed that the polarization state of macrophages was associated with changes in cell shape, and elongation could lead to upregulation of M2 phenotype marker expression [38]. However, Lv et al. showed that M1 macrophages had higher mechanical flexibility and could take up SARS-CoV-2 more readily by deformation [14]. Therefore, the morphological changes in MH-S cells reflected that rLLB exposure may cause the polarization of macrophages. However, the mechanism of phenotype transformation of AMs by rLLB exposure is not well understood. This study reported that AMs were converted to the M1 phenotype after rLLB treatment. The expression of M1 macrophage-related molecules iNOS, CD86, and IL-6 was significantly upregulated, and the expression of M2 macrophage-related molecules Arg1, CD206, and IL-10 was downregulated or not significantly changed. It also suggested that the conversion of M1 and M2 is a dynamic and continuous process. This was consistent with previous research showing that if macrophages were subjected to a combination of pro-inflammatory and pro-healing stimuli (LPS, interferon (IFN)-γ, IL-4, and IL-13), mouse macrophages acquired a mixed activation state, with individual cells expressing both M1 biomarker CD86 and M2 biomarker CD206, rather than polarizing to a discrete phenotype [39,40]. After rLLB treatment, it was found that macrophages may polarize to the M1 phenotype and M2 phenotype simultaneously and perform their respective pro-inflammatory or anti-inflammatory functions. However, overall, they were in a pro-inflammatory state of polarization toward the M1 phenotype. This may be an instinctive response of macrophages to resist external stimuli. The pro-inflammatory M1 facilitates its clearance of external stimuli. In addition, when we investigated the polarization of MH-S cells at different time points after rLLB treatment, we found that the expression of macrophage polarization-related molecular markers showed a prophase polarization to the M1 phenotype, and then returned to the normal level at 48 h. These results suggested that the phenotypic transformation of macrophages induced by rLLB waves does not fully conform to the traditional M1/2 macrophage phenotype paradigm, but more reflected the high plasticity of macrophages and the core function of regulating the maintenance of immune homeostasis. 

Macrophage polarization usually correlates with altered oxidative stress status and apoptosis [41]. Therefore, we further investigated the effects of rLLB exposure on oxidative stress and apoptosis. The results showed that rLLB exposure caused ROS burst in MH-S cells. ROS are mediators of intracellular signaling cascades that induce intracellular Ca^2+^ accumulation and the collapse of the mitochondrial membrane potential, thereby triggering apoptosis [42]. Therefore, we further examined the changes in intracellular Ca^2+^ concentration. The results showed that rLLB treatment did cause intracellular Ca^2+^ accumulation. Chronologically speaking, this event occurred after the ROS burst. Likewise, we detected a decrease in mitochondrial membrane potential, which is an early phenomenon of apoptosis. Further detection of apoptosis by AnnexinV/PI staining revealed that rLLB exposure only had a more pronounced effect on early apoptosis. Meanwhile, apoptotic pathway upstream molecules Bax and Bcl2 and executive protein cleaved-Caspase3 had almost no change. It indicates that rLLB exposure only caused early apoptosis of AMs and not late apoptosis, probably due to the low intensity of the blast wave. ROS are originally important mediators produced by activated macrophages for killing pathogens [43]. However, our study found that ROS induced early apoptosis of alveolar macrophages by triggering mitochondrial dysfunction. For rLLB to induce ROS production but only cause early apoptosis, it may be related to the lower intensity of the shock wave, or it may be associated with the short duration of ROS. That is, ROS peaked only at 12 h and decreased significantly at 24 h. Under normal physiological conditions, alveolar macrophages have a long lifespan as tissue-resident cells [44] and play an important role in phagocytosis, immunity, and secretion. However, rLLB exposure could cause early apoptosis of AMs, causing them to enter the programmed death phase early and affecting their normal physiological functions and proliferation, which was also a reflection of the imbalance of immune homeostasis caused by rLLB exposure.

Previous studies of U.S. troops serving during the war in Afghanistan have demonstrated that rLLB exposure could trigger post-traumatic stress disorder (PTSD)-like symptoms, such as depression, impulsivity, anxiety, and insomnia [7]. It also caused concussion-like symptoms such as headache, slowed thinking, memory loss, and cognitive impairment [8] and affected hearing and other aspects [9]. Our study complements the findings of potential harm of rLLB exposure on the lung and the immune microenvironment of the organism. The recently enacted FY2020 National Defense Authorization Act (NDAA) stipulates that military personnel wear pressure transducers during training and combat to monitor the level of blast wave exposure [45]. Combined with clinical data from these personnel, it would be important to assess the hazards of occupational exposure to rLLB.

The Akt/NF-κB signaling pathway plays an essential role in macrophage phenotype transformation [25,46,47]. For example, Tang et al. found that TPL reduced macrophage infiltration and M1 polarization by inhibiting the PDE4B/Akt/NF-κB signaling cascade reaction in dextran sulfate sodium (DSS)-induced murine experimental colitis in vitro and in vivo [48]. The present study results were similar to the above results in that rLLB exposure promoted the conversion of AMs to the M1 phenotype by activating the Akt/NF-κB signaling pathway. In contrast, the addition of either GDC-0068 (an inhibitor of Akt) or PDTC (an NF-κB inhibitor) inhibited the polarization of AMs to M1 phenotype, suggesting that the Akt/NF-κB signaling pathway played a vital role in this. That is to say, although new cell therapy technologies for lung injury have progressed rapidly in recent years [49]. But targeting the Akt/NF-κB signaling pathway may be a potential therapeutic target for suppressing rLLB-induced inflammatory responses. The development of inhibitors, drugs or antibodies against this pathway may have good therapeutic results. This study is expected to provide molecular indicators for early diagnosis of immune homeostasis imbalance triggered by rLLB exposure and provides potential molecular targets for future prevention and treatment of related diseases.

To the best of our knowledge, this study is the first to introduce a miniature manual Reddy-tube device into the study of blast injury on AMs. The model has the advantage of being simple and easy to operate in an ultra-clean table, addressing the requirement of sterility for in vitro models of blast injury. To simulate rLLB exposure at the cellular level, we modified the Reddy-tube device by increasing the number of septum layers to reach a peak overpressure of 0.78 bar and simulating rLLB exposure by repeatedly applying shock waves. It provided a new, reliable and easy-to-use modeling method for the much-needed in vitro study of molecular mechanisms in the field of rLLB exposure. However, the current research only investigated the effects of rLLB exposure on a murine alveolar macrophage cell line, MH-S cells. In principle, further investigation in vivo and in primary alveolar macrophage cells is needed. However, primary alveolar macrophage cells are sensitive, and the isolation and sorting process for primary cells involves various biomechanical factors. Beyond these problems, the rLLB-related research on primary alveolar macrophage cells may be more in-depth and meaningful. The dilemma of in vivo rLLB animal models is that the shock wave needs to pass through the thorax or airway to reach the lungs. The shock wave conducts in a complex environment in the alveoli, and the rLLB has low intensity. The real shock wave overpressure on alveolar macrophages in vivo cannot be accurately measured under the current experimental conditions. Differences in low overpressure intensity may be one of the reasons why people in different occupations have different adverse reactions. In contrast, the in vitro cell model can relatively accurately measure the shock wave overpressure intensity and time acting on the cell surface, laying a solid foundation for in vivo experiments.

## 4. Materials and Methods

### 4.1. Cell Culture and Grouping

The murine alveolar macrophage cell line MH-S (ATCC, CRL-2019) was cultured in RPMI-1640 containing 10% FBS and antibiotics (100 IU/mL penicillin and 0.1 mg/mL streptomycin, P1400, Solarbio, Beijing, China), with the culture conditions of 37 °C and humid air containing 5% CO_2_. MH-S cells were divided into two groups: normal control (NC) group and repetitive low-level blast treatment (rLLB) group, and collected at 6 h, 12 h, 24 h, 36 h, and 48 h for assay; or into four groups: NC, rLLB, rLLB plus 10 μM inhibitor (rLLB + GDC, or rLLB + PDTC) group, and inhibitor (10 μM, GDC or PDTC) group, and collected at 24 h for assay. Akt inhibitor (GDC-0068, APExBIO, Houston, TX, USA) and NF-κB inhibitor (PDTC, Beyotime, Haimen, China) were added 2 h before cell collection to assess their inhibitory effects on signaling pathways.

### 4.2. Miniature Manual Reddy-Tube Device for rLLB Generation

Cells were treated with rLLB waves generated by a modified miniature manual Reddy-tube device [50] as shown in Figure 1. Briefly, the device consists of a syringe, two adapters, a shim, two Parafilm M membranes, and a T25 flask. A 10 mL syringe acts as the driving section (the length of the driving section is 3.4 cm). Two Parafilm M (Bemis) membranes act as septa, which are compressed by adapter 1 and the shim. They were connected to the T25 cell culture flask by adapter 2. The end of adapter 2 acts as the driven section (the length of the driven section is 1 cm). When the cell density reached 80%, we removed the medium and filled the T25 cell culture flask with phosphate buffer (PBS). A miniature manual Reddy-tube device was connected to the cell culture flask, and the syringe was pushed by a manually operated piston to compress the air in the driving section to break through the septum, generating a shock wave to the cells in the flask. This was repeated 10 times to simulate rLLB exposure. The time between each shock wave treatment was about 2 min due to the experimental operation. Peak overpressure duration was less than 1 ms. For the NC group, the Reddy-tube device was installed using the same procedure but without the addition of Parafilm, and no shock wave was generated.

### 4.3. Observation of Cell Morphology and Calculation of Morphological Change Rate and Aspect Ratio

MH-S cells were treated with rLLB 10 times and samples collected at 6 h. Cell morphological changes were observed using an optical microscope. Cells with regular edges and shiny circles under the microscope were defined as normal cells; cells that lost their regular circular shape and protruded pseudopods were defined as morphologically altered cells [51]. The numbers of regular and morphologically altered cells in the NC and rLLB groups were counted to calculate the morphological alteration rate of MH-S cells induced by rLLB exposure. The longest and shortest diameters of the cells in the NC and rLLB groups were measured by ImageJ software, and the cell aspect ratio was calculated. The changes in the cell aspect ratio were used to reflect the degree of morphological changes.

### 4.4. Cytoskeleton Staining

After treatment with rLLB for 6 h, MH-S cells were fixed with 4% paraformaldehyde (P1110, Solarbio, Beijing, China), then YF^®^488-phalloidin (#YP0059, US EVERBRIGHT, Beijing, China) and Alexa Fluor^®^ 594-β-Tubulin (#KM9003T, SUNGENE, Tianjin, China) were applied to stain microfilaments and microtubules, respectively. Moreover, cells were re-stained with 4′,6-diamidino-2-phenylindole (DAPI) (C0060, Solarbio, Beijing, China) to show the nuclei, and finally, images were acquired using a confocal microscope (A1, Nikon, Tokyo, Japan).

### 4.5. Real-Time Quantitative PCR (qPCR)

TRIzol reagent (#19201ES60, YEASEN, Shanghai, China) was used to lyse cells and extract total RNA. The purity and concentration of RNA were determined by NanoDrop One (Thermo Fisher Scientific, Waltham, MA, USA). Hifair^®^ II 1st Strand cDNA Synthesis Kit (gDNA digester plus) (#11139ES60, YEASEN, Shanghai, China) was used for reverse transcription, and Hieff^®^ qPCR SYBR Green Master Mix (No Rox) (#11201ES03, YEASEN, Shanghai, China) was used for qPCR. Detection was performed using a qPCR instrument (LightCycler^®^ 96, Roche, Indianapolis, IN, USA). We used the 2^−ΔΔCt^ method to calculate the relative expression levels. The primers were synthesized by GenScript Biotech Corporation, and the primer sequences are shown in Table 1.

### 4.6. Western Blotting (WB) Analysis

Proteins were obtained from MH-S cells using ice-cold RIPA Lysis Buffer (#20101ES60, YEASEN, Shanghai, China) with protease inhibitor and phosphatase inhibitor added. Total protein (40 μg) was subjected to SDS-PAGE electrophoresis and then transferred to PVDF membranes. After blocking with 5% skimmed milk, the membranes were probed with primary antibodies against iNOS (1:1000, # AF0199, Affinity, Liyang, Jiangsu, China), CD86 (1:500, #sc-28347, Santa Cruz, Santa Cruz, CA, USA), IL-6 (1:500, #sc-57345, Santa Cruz, CA, USA), Arg1 (1:5000, #16001-1-AP, Proteintech, China), CD206 (1:500, #sc-58986, Santa Cruz, CA, USA), IL-10 (1:500, #sc-365858, Santa Cruz, USA), Bax (1:1000, #AF0120, Affinity, Liyang, China), Bcl2 (1:1000, #AF6139, Affinity, Liyang, China), Caspase3 (1:1000, #AF6311, Affinity, Liyang, China), Cleaved-caspase3 (1:1000, #AF7022, Affinity, Liyang, China), Akt (1:1000, #AF6261, Affinity, Liyang, China), p-Akt (Ser473) (1:1000, #AF0016, Affinity, Liyang, China), NF-κB p105/p50 (1:1000, #AF6217, Affinity, China), NF-κB p-p105/p50 (Ser337) (1:1000, #AF3219, Affinity, Liyang, China), NF-κB p65 (1:1000, #AF5006, Affinity, Liyang, China), NF-κB p-p65 (Ser536) (1:1000, #AF2006, Affinity, China), GAPDH (1:5000, K200103M, Solarbio, Beijing, China) and β-Tubulin (1:5000, #KM9003T, SUNGENE, China) at 4 °C overnight. After being washed with PBS containing Tween 20 (PBST), the PVDF membranes were incubated with HRP-coupled secondary antibodies (1:5000, #ZB-2301 and ZB-2305, ZSGB-BIO, Beijing, China) at room temperature for 1 h. ECL Chemiluminescent Reagent (#36208ES60, YEASEN, Shanghai, China) was used to display protein bands. Tanon 5200 Multi detection system obtained images, and the Tanon Gel-Pro Analyzer system analyzed the intensity of each band.

### 4.7. Immunofluorescence

After discarding the medium, MH-S cells were washed twice with ice-cold PBS. Cells were subsequently fixed with 4% paraformaldehyde at room temperature for 20 min and then permeated with PBS containing 0.5% Triton X-100 for 20 min. The cells were then blocked with PBS solution containing 3% BSA for 1 h at room temperature, then incubated with primary antibodies against iNOS (1:100), Arg1 (1:100), p-Akt (1:100), p-p105 (1:100), and p-p65 (1:100) overnight at 4 °C, respectively. After washing, cells were incubated with Alexa Fluor^®^ 488 or Alexa Fluor^®^ 594-labeled fluorescent secondary antibodies of the corresponding species (1:1000, ZB-0511 and ZB-0513, ZSGB-BIO, China) for 1 h at room temperature protected from light. DAPI was incubated for 3 min for nuclear staining. Finally, images were acquired using an inverted fluorescence microscope (Ts2R, Nikon, Tokyo, Japan).

### 4.8. ROS Staining and Mitochondrial Membrane Potential Detection

MH-S cells culture medium was discarded, and cells were washed twice with ice-cold PBS. 2′, 7′ dichlorodihydrofluorescein diacetate (DCFH-DA, #D6470, Solarbio, Beijing, China) was applied as a fluorescent probe to detect intracellular ROS levels by co-incubation with cells for 20 min in a 37 °C cell incubator. JC-1 (#M8650, Solarbio, Beijing, China) was applied as a fluorescent probe to detect the change in intracellular mitochondrial membrane potential (ΔΨm) by co-incubation with cells for 20 min at 37 °C in a cell incubator. An inverted fluorescence microscope was used to observe the experimental results.

### 4.9. Flow Cytometry

After the MH-S cells were treated according to experimental groupings, 1×10^6^ cells were collected by centrifugation, resuspended in PBS, and assayed using a CytoFLEX flow cytometer (Beckman Coulter, Brea, CA, USA). Changes in intracellular Ca^2+^ concentration were detected using Fluo-4 AM Cell Membrane Permeable Calcium Ion Fluorescent Probe (#40704ES50, YEASEN, Shanghai, China). Cells were co-incubated with Fluo-4 AM for 30 min in a 37 °C incubator, and washed with HBSS solution 3 times. HBSS solution was added to cover the cells, incubated for 30 min in a 37 °C incubator, and then flow cytometry detection was performed. Apoptosis was detected using Annexin V-FITC/PI Apoptosis Detection Kit (#40305ES20, YEASEN, Shanghai, China). Cells were incubated with Annexin V-FITC and PI Staining Solution at room temperature for 15 min in the dark, then mixed with Binding Buffer and placed on ice, followed by the assay on the flow cytometer. Macrophage polarization was detected by CD86 and CD206 antibodies that labeled M1 or M2 macrophages, respectively. Cells were co-incubated with anti-CD86 and anti-CD206 antibodies at room temperature for 30 min and washed with PBS, then incubated with Alexa Fluor^®^ 488/594-coupled secondary antibodies for 30 min at room temperature and then assayed on the flow cytometer.

### 4.10. Cell Viability Assay

MH-S cells were inoculated at a density of 5 × 10^3^ cells/well in 96-well plates (701001, NEST Biotechnology Co. Ltd., Wuxi, China) for 24 h. The cells were treated with different concentrations of GDC-0068 (1, 5, 10, 20, 40, 60, 80, 150, and 200 μM) for 12 h. Then, the cells were washed with PBS and co-incubated with CCK-8 solution (#40203ES60, YEASEN, Shanghai, China) in a 37 °C incubator for 1 h. The Microplate Reader (Hangzhou Allsheng Instrument Co., Ltd., Hangzhou, China, FlexA-200) was used to detect the optical density (OD) values at 450 nm.

### 4.11. Statistical Analysis

Continuous variables with normal distribution were used as mean ± standard deviation (SD). Two-group comparisons of continuous variables were performed using unpaired t-tests. The results for multiple time points in the NC and rLLB groups were analyzed using two-way ANOVA followed by Sidak’s multiple comparisons tests. The results for the four subgroups were analyzed using one-way ANOVA followed by Tukey’s multiple comparisons. Statistical analysis and graphing were performed using GraphPad Prism 8.0 software. Significance levels were set as follows: * *p* < 0.05; ** *p* < 0.01; *** *p* < 0.001, ns: no significance. Each experiment was repeated at least three times.

## 5. Conclusions

The current study suggests that rLLB exposure can cause morphological alterations and polarization toward the M1 phenotype of AMs, and rLLB exposure can also cause altered oxidative stress status and early apoptosis in AMs. The underlying molecular mechanism of M1 polarization is the Akt/NF-κB signaling pathway (Figure 8). Although further validation is needed, our preliminary data may provide a potential new therapeutic strategy for preventing and mitigating the hazards of rLLB exposure in occupational populations.

## Figures and Tables

**Table 1:** qPCR primer sequences.

Gene Names	Forward Primer (5′-3′)	Reverse Primer (5′-3′)
*iNOS*	GTTCTCAGCCCAACAATACAAGA	GTGGACGGGTCGATGTCAC
*CD86*	TGTTTCCGTGGAGACGCAAG	TTGAGCCTTTGTAAATGGGCA
*IL-6*	CCAAGAGGTGAGTGCTTCCC	CTGTTGTTCAGACTCTCTCCCT
*Arg1*	CTCCAAGCCAAAGTCCTTAGAG	AGGAGCTGTCATTAGGGACATC
*CD206*	CTCTGTTCAGCTATTGGACGC	CGGAATTTCTGGGATTCAGCTTC
*IL-10*	GCTCTTACTGACTGGCATGAG	CGCAGCTCTAGGAGCATGTG
*Bax*	AGGCGAATTGGCGATGAACTGG	CTAGCAAAGTAGAAAAGGGCAACC
*Bcl2*	ATGCCTTTGTGGAACTATATGGC	GGTATGCACCCAGAGTGATGC
*Caspase3*	TGGTGATGAAGGGGTCATTTATG	TTCGGCTTTCCAGTCAGACTC
*Akt*	ATGAACGACGTAGCCATTGTG	TTGTAGCCAATAAAGGTGCCAT
*p105*	ATGGCAGACGATGATCCCTAC	TGTTGACAGTGGTATTTCTGGTG
*p65*	AGGCTTCTGGGCCTTATGTG	TGCTTCTCTCGCCAGGAATAC
*GAPDH*	AGGTCGGTGTGAACGGATTTG	TGTAGACCATGTAGTTGAGGTCA

## Data Availability

The original contributions presented in the study are included within the article. Further inquiries can be directed to the corresponding author.

## Abbreviations

AMs	Alveolar macrophages
Arg1	Arginase 1
ARDS	Acute respiratory distress syndrome
BOP	Blast overpressure
CD	Cluster of differentiation
COPD	Chronic obstructive pulmonary disease
CCK-8	Cell counting kit-8
DSS	Dextran sulfate sodium
FBS	Fetal bovine serum
HLB	High-level blast
HBSS	Hank’s balanced salt solution
HRP	Horseradish peroxidase
IL	Interleukin
iNOS	Inducible nitric oxide synthase
IC_50_	Half maximal inhibitory concentration
IFN	Interferon
LPS	Lipopolysaccharide
NC	Normal control
NDAA	National Defense Authorization Act
NF-κB	Nuclear factor kappa-B
OD	Optical density
PVDF	Polyvinylidene fluoride
PTSD	Post-traumatic stress disorder
qPCR	Real-Time Quantitative PCR
rLLB	Repetitive low-level blast
ROS	Reactive oxygen species
SD	Standard deviation
TNF	Tumor necrosis factor
TGF	Transforming growth factor
TBI	Traumatic brain injuries
TPL	Triptolide
WB	Western blotting
